# Glucuronyl Glucosyloleanolate: A Safe and Potent AI‐Discovered Depigmenting Saponin

**DOI:** 10.1111/jocd.71210

**Published:** 2026-09-23

**Authors:** Yannick Quesnel, Michael Gold

**Affiliations:** ^1^ Kokuma Charleroi Belgium; ^2^ Gold Skin Care Center, Tennessee Clinical Research Center Nashville Tennessee USA

## Abstract

**Purpose:**

Saponins are amphiphilic glycosides found in plants. They have long been used in traditional Asian remedies for their anti‐inflammatory, protective, and rejuvenating properties. One representative, Glucuronyl Glucosyloleanolate (GGO), was recently discovered using a combination of artificial intelligence and experimental testing as a depigmenting agent. Its safety has been verified in humans during a patch test on 12 volunteers, followed by an HRIPT study on 100 volunteers. Finally, its depigmenting efficacy has been demonstrated after a clinical trial on 60 volunteers.

**Methods:**

- A 48‐h patch test was conducted on 12 volunteers using the pure compound.
- HRIPT: a patch with 0.1% GGO in mineral oil was applied to 100 volunteers repeatedly over 3 weeks. After a 2‐week rest, a single patch was applied again to the induction site and to a new, untreated site for the challenge phase.
- Clinical trial: monocentric blind study of GGO (0.01% weight) versus placebo on a multi‐ethnic panel with pigmentation spots.

**Results:**

- Safety: No skin irritation or adverse effects were observed in the patch test assay confirming the product's excellent safety. On the 100 volunteers, during the induction period, no reaction of irritation was observed after 3 weeks and during the challenge phase, no allergic reaction was observed. Based on these results, GGO demonstrated very good skin compatibility and does not show a sensitizing effect.
- Efficacy: all subgroups showed significant depigmentation from Day 21 onwards, which continued to increase in a similar manner to that observed with localized depigmentation. After 84 days, the level of depigmentation achieved was 10% on Asian, 14% on Caucasian and North African skin, and 24% on black skin. Dark Pigmentation spots on Asian and Caucasian skin depigmented at a rate 34% faster than the overall skin tone, resulting in their visual fading

**Conclusion:**

Glucuronyl Glucosyloleanolate, identified through AI‐enhanced biopharma strategies, shows strong skin‐brightening efficacy and safety. It depigments dark spots faster than skin tone brightening in an 84‐day clinical trial. Non‐irritant and potent at low doses, it promises broad cosmetic and medical applications (melasma). Future investigations should assess its anti‐aging and antioxidative potential to broaden its scope of application.

## 1 Introduction

Saponins are amphiphilic plant glycosides known for their surfactant and protective roles in plants, whose biological activities have long been exploited in Asian traditional medicine. Extracts rich in saponins are widely used for their anti‐inflammatory, protective, and rejuvenating effects. One such molecule, Glucuronyl Glucosyloleanolate (GGO), was recently identified through AI‐guided discovery combined with experimental validation as a promising depigmenting agent. The discovery pipeline followed a rigorous stepwise approach, progressing from computational screening to in vitro, ex vivo, and finally in vivo validation, ensuring that only the most promising and safest candidate advanced to clinical testing.

Safety was first confirmed by a patch test at high concentration on 12 volunteers, followed by a Human Repeated Insult Patch Test (HRIPT) involving more than 100 participants. Both studies confirmed excellent skin compatibility, with no irritation or sensitization observed. Depigmenting and brightening efficacy was then assessed in a randomized, single‐blind, placebo‐controlled clinical study, where significant depigmentation was observed across all ethnic subgroups from Day 21, with progressive improvement up to Day 84. Depigmentation reached 10% in Asian subjects, 14% in Caucasian and North African subjects, and 24% in Black subjects. Moreover, dark spots on Asian and Caucasian skin faded 34% faster than overall skin tone, a kinetic profile that distinguishes GGO from most currently available depigmenting agents.

Overall, GGO appears to be a safe and potent skin‐brightening compound active at remarkably low doses, offering strong potential for both cosmetic and medical applications, including the treatment of hyperpigmentation disorders such as melasma, post‐inflammatory hyperpigmentation, and age spots.

## 2 Material and Methods

### 2.1 Patch Test

A patch test was performed under dermatological supervision and in accordance with the Declaration of Helsinki (1964), following Independent Ethics Committee approval. All subjects provided written informed consent prior to participation. Pure GGO was applied on the back (scapular zone) of 12 volunteers (7 women, 5 men; aged 20–70) as a semi‐occlusive patch (with filter paper) for 48 h. Erythema and oedema were scored by a qualified physician at study completion, using standardized grading criteria. The Mean Irritation Index (MII) was calculated as the primary endpoint.

### 2.2 HRIPT

The HRIPT followed major international ethics and Good Clinical Practice standards, including the Declaration of Helsinki, ICH E6(R3), EU Regulation 2014/536, Colipa guidelines, and Romanian GCP regulations after Independent Ethics Committee approval. All subjects provided written informed consent. The study was designed to determine whether GGO (diluted at 0.1% wt with mineral oil, representing ten times the active dose used in the clinical trial) causes delayed contact sensitization under maximized exposure conditions. A panel of 102 healthy adult volunteers (see Table 1) received nine semi‐occlusive patch applications on the upper back over 3 weeks (induction phase), followed by a two‐week rest period and a single challenge application on both the induction and a virgin site, with water as negative control. Skin reactions, discomfort, and any allergic responses were systematically evaluated using the International Contact Dermatitis Research Group (ICDRG) scale and reaction rates at each assessment visit.

**TABLE 1:** HRIPT on one hundred of volunteers.

Criteria	Included test subject *N* = 108		Valid cases *N* = 101	
	Nb	%	NB	%
*Phototype*				
I	1	1%	1	10%
II	28	26%	24	24%
III	70	65%	68	67%
IV	9	8%	8	8%
*Sex*				
Male	13	12%	10	10%
Female	95	88%	91	90%
**Age**	**Years**		**Years**	
Minimum	32		32	
Maximum	70		70	
Mean	56		56	

### 2.3 Clinical Trial

A randomized, single‐blind, placebo‐controlled clinical study was conducted under the supervision of a dermatologist, in accordance with Good Clinical Practices (CPMP, July 1996), French law n°2001–806 (August 2004), and the Declaration of Helsinki (1964). All subjects provided written informed consent prior to enrolment. A total of 60 volunteers were randomized into two groups of 30: the GGO treatment group (Group A, 0.01% GGO in cream formulation) and the placebo group (Group B, identical formulation without GGO). Each group was further stratified by skin phototype and ethnic origin as follows:

- 10 participants of African or North African origin (phototype IV–V)
- 10 of Asian origin (phototype III‐IV)
- 10 of Caucasian origin (phototype III)

Participants applied their assigned product twice daily (after morning and evening hygiene routines) for 84 days. To prevent UV‐related confounding, all volunteers applied SPF_50+_ sunscreen (Avène) during daylight hours throughout the study period. Clinical assessments were conducted at four time points: Day 0, Day 21, Day 56, and Day 84. The following evaluations were performed at each visit:

- Measurement of ITA° (Individual Typology Angle) using a Chromameter CR400 (Konica Minolta) as a validated objective measure of skin lightening.
- 2D facial photography using the VISIA imaging system, enabling quantitative assessment of hyperpigmentation spots.

## 3 Results

A 48‐h patch test on 12 volunteers using pure GGO produced no skin irritation or adverse effects. The Mean Irritation Index (MII) was 0 (Table 2), a value well below accepted thresholds, leading to the unambiguous classification of the compound as non‐irritant.

**TABLE 2:** 2 days GGO patch test assay on 12 volunteers.

N°	Age	Sex	Phototype	Result 48 h				
				Blank		GGO		MII
				Erythema	Oedema	Erythema	Oedema	
1	65	M	III	Neg	Neg	Neg	Neg	0
2	67	F	III	Neg	Neg	Neg	Neg	0
3	46	F	III	Neg	Neg	Neg	Neg	0
4	68	M	III	Neg	Neg	Neg	Neg	0
5	51	M	III	Neg	Neg	Neg	Neg	0
6	53	F	III	Neg	Neg	Neg	Neg	0
7	50	F	IV	Neg	Neg	Neg	Neg	0
8	38	M	III	Neg	Neg	Neg	Neg	0
9	67	F	III	Neg	Neg	Neg	Neg	0
10	27	F	IV	Neg	Neg	Neg	Neg	0
11	29	F	I	Neg	Neg	Neg	Neg	0
Conclusion: Mean Irritation index								0

*Note:* O: Neg (No erythema/No Oedema)—0.5: Slight Erythema/slight Oedema—1: Moderate Erythema/moderate Oedema—2: acute Erythema/important Oedema—3: Vivid erythema diffusing outside the tested area/Important oedema with vesicles.

This result was reinforced by the HRIPT findings (Table 3). During the induction period, nine repeated semi‐occlusive applications of GGO (0.1% in mineral oil) on 102 subjects with all skin types induced no irritation reaction in any participant. During the challenge phase, a single application on 101 subjects at both the induction and virgin sites induced no allergic reaction whatsoever. Based on these results, GGO demonstrates excellent skin compatibility with no sensitizing effect. The absence of sensitizing evidence under the experimental conditions adopted supports the marketing claim “hypoallergenic,” while not excluding the theoretical possibility of rare sensitization events in very large populations, as is standard for any topical ingredient.

**TABLE 3:** HRIPT clinical data.

Induction phase *N* = 102			
Type of reaction	Description of the reaction on the induction site	Number and %age of reactive test subjects	Total number and %age of reactive test subjects
E: Erythema	None	0/0	0/0
M: Complementary mention	None	0/0	

Challenge *N* = 101			
Type of reaction	Description of the reactions on the induction site and the virgin site	Number and %age of reactive test subjects	Total number and %age of reactive test subjects
E: Erythema	None	0/0	0/0
M: Complementary mention	None	0/0	
A: ICDRG scale	None	0/0	

**FIGURE 1:**
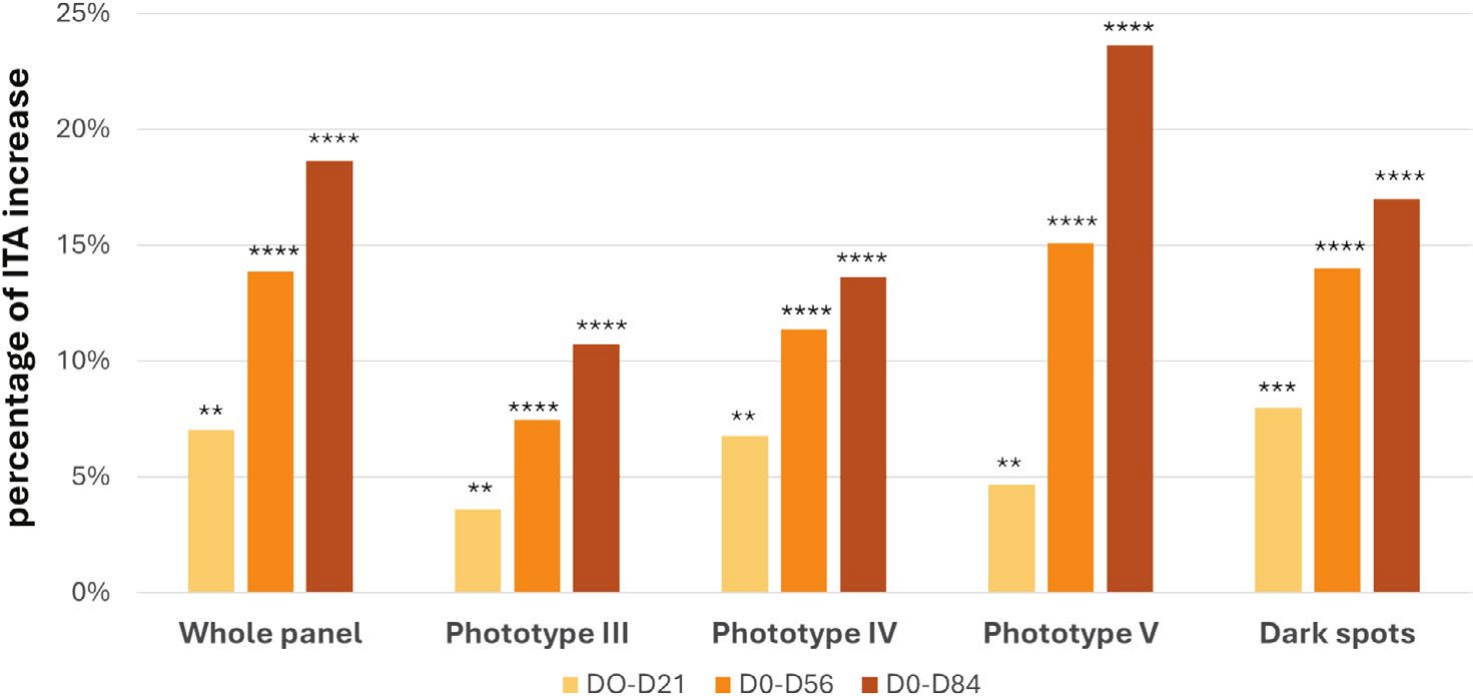
Skin tone and dark spots changes in ITA over time compared with Placebo.

GGO was subsequently evaluated in a single‐blind randomized controlled trial for overall skin brightening and reduction of hyperpigmentation spots. Skin‐tone lightening increased progressively across all ethnic subgroups throughout the 84‐day treatment period, reaching 10%, 14%, and 24% reductions in pigmentation in the Asian, Caucasian/North African, and Black subgroups, respectively, by Day 84. Importantly, no activity plateau was reached at the end of the study, suggesting that further improvement would be expected with prolonged use, a particularly encouraging finding for long‐term treatment strategies (see Supporting Information). In Caucasian and Asian subgroups specifically, analysis restricted to fair skin types (phototype III and IV) confirmed a 17% spot depigmentation after 84 days without plateau (Figure 1).

**FIGURE 2:**
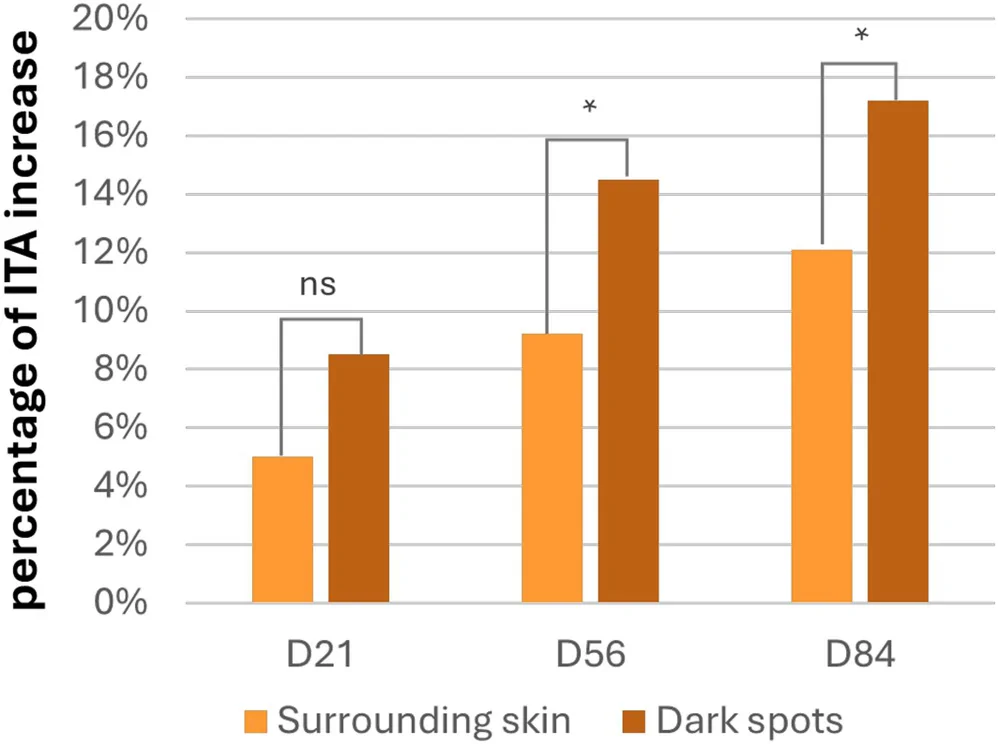
Comparative ITA changes in hyperpigmented spots and surrounding skin.

A particularly noteworthy observation was that pigmentation spots lightened significantly more rapidly than overall skin tone (Figures 1 and 2). Spot depigmentation became statistically significant as early as Day 21 and continued to improve progressively, reaching up to 17% by Day 84.

This differential kinetic, faster and more pronounced fading of localized spots relative to general skin background is uncommon among known depigmenting ingredients and constitutes a distinctive and clinically meaningful feature of GGO. In some volunteers, dark spots nearly disappeared entirely by the end of the treatment period. This property is particularly promising for dermatological applications such as melasma and post‐inflammatory hyperpigmentation, conditions that remain inadequately managed with current therapeutic options and where GGO deserves urgent further clinical investigation.

## 4 Discussion

**FIGURE 3:**
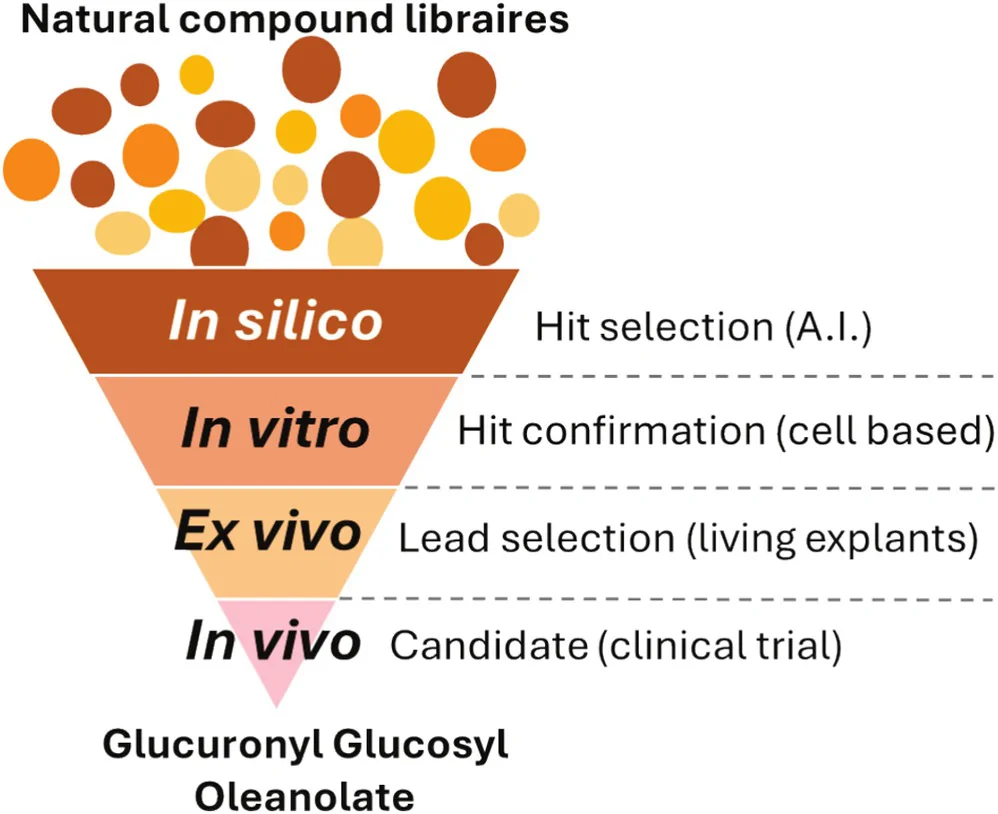
Discovery methodology which enabled GGO discovery.

GGO was discovered using a proprietary AI‐assisted virtual decision tree, and its biological activity and safety profile were confirmed through a comprehensive experimental cascade (Figure 3): in vitro testing (functional assays on human melanocytes), ex vivo testing (depigmentation assays on human skin explants), and in vivo testing (clinical safety and efficacy) [1]. This integrative strategy enables the identification of compounds meeting evolving consumer expectations for efficacy, safety, and sustainability, a field where genuine molecular innovation has slowed considerably, with much of the industry's development activity focused on incremental reformulation rather than the discovery of new active entities.

The skin depigmentation market is a rapidly growing global industry, valued at over $10 billion and expanding at 6%–8% annually, driven by aesthetic trends, cultural factors, and social media. It splits into two sectors: a regulated one using mild ingredients (vitamin C, niacinamide, tranexamic acid), and an unregulated one selling potentially dangerous products containing mercury, hydroquinone, steroids, or glutathione [2, 3]. The health risks of these unregulated products range from local skin damage (atrophy, acne, ochronosis) to serious systemic harm (liver, kidney, and neurological damage, plus increased skin cancer risk). This makes skin lightening a significant public health concern, especially where regulatory frameworks are weak, underscoring the need for safe, effective, and well‐validated alternatives.

### 4.1 Multitarget Strategy Applied to Depigmentation

**FIGURE 4:**
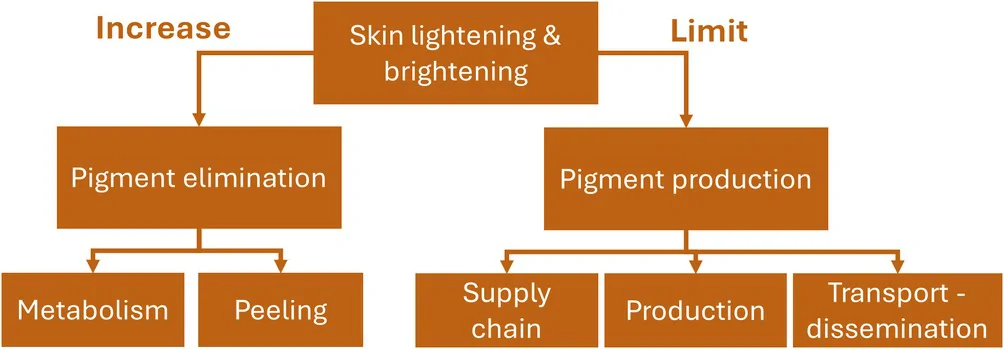
Possible strategies to tackle skin pigmentation.

Natural active ingredients acting through multiple mechanisms converging on the same physiological endpoint offer a strategic advantage in cosmetic science. This concept parallels the pharmaceutical Multitarget‐Directed Ligand (MTDL) [4] strategy, in which a single molecule modulates several biological targets simultaneously [5]. Unlike the traditional “one drug‐one target” paradigm, MTDLs exploit the complexity of interconnected physiological pathways to achieve synergistic effects, improving overall efficacy while reducing the doses required for each individual mechanism and consequently improving the safety margin. With regard to skin lightening and brightening, as shown in Figure 4, two potentially complementary strategies are used: firstly, accelerating epidermal renewal, which will speed up the disappearance of skin pigments (this is the mechanism of action of vitamin C, vitamin E and natural organic acids such as alpha hydroxy acids and salicylic acid) [6]; or limiting pigments within the different layers of the skin.

#### 4.1.1 Supply

Melanin synthesis can be limited by reducing availability of L‐tyrosine, the key biosynthetic precursor, either through dietary modification or through reactive compounds that intercept synthesis intermediates, such as glutathione (GSH) [7] or 2‐MNG (Melasyl from L'Oréal) [8].

#### 4.1.2 Production

The most direct approach to reducing pigmentation involves inhibiting intrinsic melanin biosynthesis. This can be achieved either by directly inhibiting tyrosinase enzymatic activity (the mechanism of hydroquinone, kojic acid, alpha‐arbutin, tranexamic acid, and thiamidol, several of which now face increasing regulatory scrutiny) or, in a previously unexplored mechanism, by reducing the expression level of enzymes involved in melanogenesis. GGO significantly downregulates the expression of tyrosinase, TRP1, and TRP2, as demonstrated by immunostaining in ex vivo human skin explant experiments [1]. Critically, this downregulation is not attributable to direct enzymatic inhibition of tyrosinase, representing a genuinely novel mechanism of action within the depigmentation field.

#### 4.1.3 Transport

Once produced, melanin is packaged into melanosomes and transferred from melanocytes in the basal layer to keratinocytes via dendritic contacts, enabling its distribution through the overlying skin layers. Interfering with this transfer process limits the spread of pigmentation independently of melanin synthesis. GGO is known to interfere with NF‐κB signaling [9], a pathway involved in melanosome transfer from melanocytes to keratinocytes, thereby limiting melanin propagation to the upper layers of the epidermis and contributing to an overall brightening effect. Niacinamide [10] and Rhododendrol are known to operate through related mechanisms [11].

The combination of reduced melanogenic enzyme expression and inhibition of melanosome transfer positions GGO as a genuine dual‐mechanism MTDL for depigmentation [12]. This synergistic profile was confirmed quantitatively at each step of the experimental cascade: an 86% reduction in melanin synthesis in vitro on human melanocytes, up to 45% reduction ex vivo on human skin explants [1], and up to 24% depigmentation of overall skin tone and 17% spot fading in vivo during the clinical trial (Table 1), remarkable outcomes for a single compound used at a concentration as low as 0.01% (Table 4).

**TABLE 4:** Main depigmenting agents, MoA and loading in final product.

Active Ingredient	MoA	Strategy	Loading
Hydroquinone	Tyrosinase inhib.	Production	1%–5%
Kojic acid	Tyrosinase inhib.	Production	1%
Niaciamide	Melanosome transfer	Transport	Up to 20%
Alpha‐arbutine	Tyrosinase inhib.	Production	0.5%
Tranexamic acid	Tyrosinase inhib.	Production	5%
Thiamidol	Tyrosinase inhib	Production	0.5%
2‐MNG	Reactive species	Supply	0.5%
GGO	Tyr‐ expression + Melanosome transfer	Production + transport	0.01%

The safety and tolerance profile was equally strong. On human skin explants, full tissue structure and integrity were preserved throughout the experiment, a meaningful outcome, since some tyrosinase inhibitors are known to compromise skin integrity in comparable test models. Combined with the patch test (MII = 0.04) and HRIPT (zero reactions in over 100 subjects), GGO is firmly classified as both non‐irritant and non‐sensitizing, confirming its suitability for broad topical use across diverse skin types and phototypes.

### 4.2 GGO In the Context of the Saponin Family

Glucuronyl Glucosyloleanolate belongs to the saponin family [13], a chemically diverse subclass of secondary plant metabolites derived from steroids or triterpenes. Structurally, saponins consist of a hydrophobic aglycone linked to one or more hydrophilic sugar chains, conferring amphiphilic character and strong surfactant properties. In the plant kingdom, saponins serve as protective compounds, deterring herbivores through their bitter taste and exhibiting antimicrobial and antifungal activities [14, 15]. These properties have inspired numerous industrial applications, from natural detergents and food emulsifiers to agricultural biopesticides and vaccine adjuvants [16, 17, 18, 19].

**FIGURE 5:**
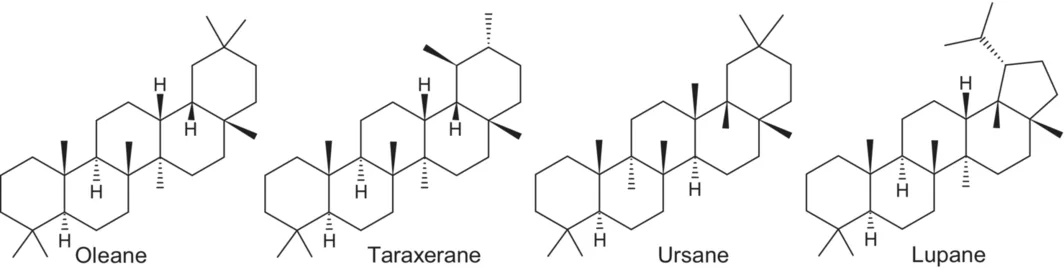
Major triterpenoid cores found into saponins.

Among the four major triterpene saponin families: oleanane, taraxerane, ursane, and lupane (Figure 5) [20], the oleanane scaffold and specifically oleanolic acid was of particular interest [21]. Oleanolic acid has been identified in more than 2000 plant species, making it one of the most widely distributed triterpenes in the plant kingdom. Its broad availability and high content in certain extractable plant sources ensure supply at commercially viable cost, enabling further chemical derivatization to amplify its beneficial properties. Oleanolic acid is relatively non‐toxic and exhibits multiple well‐documented biological activities including anticancer [22], antiviral [23], antibacterial [24], hepatoprotective [25, 26], neuroprotective [27], antidiabetic [28], anti‐obesity [29], antioxidant [30], and anti‐inflammatory effects [31].

**FIGURE 6:**
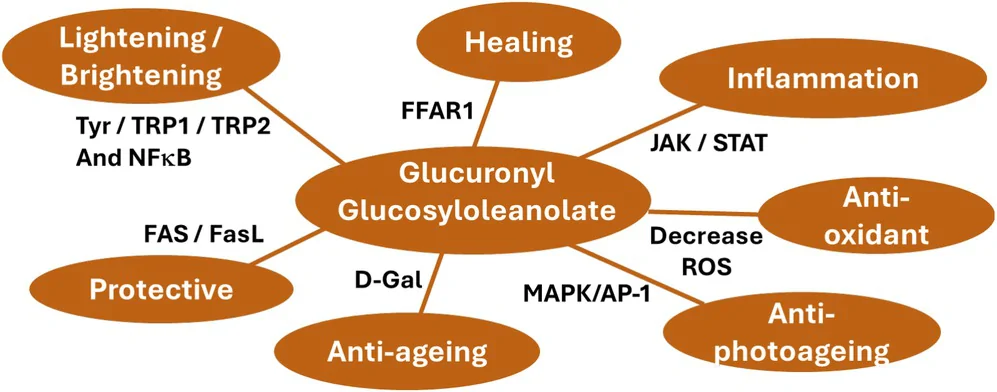
Demonstrated and reported a beneficial effect of GGO on skin.

As a close metabolic derivative of oleanolic acid, GGO shares and extends this broad pattern of biological activity (Figure 6). Published data demonstrate GGO's pharmacological relevance in oncology (via PI3K/Akt, PKCα‐ERK1/2, TLR4/NF‐κB, and Wnt/β‐catenin signaling pathways via MMP2, MMP9, and Caspase‐3) [32], diabetes (FFAR1 activation) [33], inflammation (JAK/STAT pathway) [34], and fibrosis [35]. In the dermatology and cosmetics fields specifically, GGO has demonstrated anti‐inflammatory (via ROS reduction) [36], wound‐healing (via FFAR1 activation) [37], protective (via Fas/FasL pathway) [38], anti‐aging (via improvement of autophagy and mitophagy) [39], and anti‐photoaging (via MAPK/AP‐1 modulation) [40] activities, a constellation of skin benefits that complements and reinforces the potent depigmenting properties demonstrated in the present work.

Saponins have deep roots in Asian traditional medicine, where their biological effects were empirically recognized centuries before their chemical nature was understood. In Traditional Chinese Medicine, saponin‐rich plants such as ginseng ( *Panax ginseng* ), astragalus (*Astragalus membranaceus*), and licorice (*Glycyrrhiza uralensis*) are used to strengthen immunity, enhance endurance, reduce fatigue, and support systemic recovery [21]. In Ayurveda, *Asparagus racemosus* (Shatavari) is valued for its rejuvenating and adaptogenic properties, while soapnut serves as a natural cleanser. Across East and Southeast Asia, *Centella asiatica* and *Camellia sinensis* are appreciated for their circulatory and anti‐inflammatory benefits. This long‐standing traditional heritage is now increasingly supported and rationalized by modern pharmacological and mechanistic evidence.

### 4.3 Single Molecule vs. Ingredient Cocktails

A key advantage of a single multitarget molecule over formulation cocktails lies in the absence of ingredient‐ingredient interactions. Combination therapies, widely employed by the cosmetics industry, carry risks related to pharmacokinetic incompatibilities and potential additive toxicity between co‐formulated actives [41]. By contrast, a single MTDL such as GGO delivers synergistic mechanistic effects within a simpler, more predictable, and safer toxicological profile [42]. Furthermore, by restricting innovation to incremental reformulation of historically available ingredients accessible to all market players, the industry deprives consumers of genuinely disruptive solutions. GGO exemplifies what rigorous, AI‐assisted scientific research can deliver: a unique compound acting through multiple convergent and complementary mechanisms, offering effective and safe solutions in a pigmentation market that has long lacked true molecular innovation.

## 5 Conclusion

Natural products are gaining increasing importance in the cosmetics industry, driven by consumer demand for transparency, sustainability, and proven efficacy. GGO was discovered through an approach combining artificial intelligence and an experimental decision tree and belongs to the saponin family, well known for its diverse beneficial properties and its deep integration into Asian traditional medicine. Its skin benefits extend beyond depigmentation of spots and brightening of skin tone to encompass anti‐aging, protective, anti‐inflammatory, and healing properties, making it a genuinely multifunctional active ingredient.

GGO demonstrated outstanding safety and clinical efficacy across a rigorously conducted three‐stage validation program:

- No irritation following a 48‐h semi‐occlusive patch test with the pure compound on 12 volunteers.
- Complete absence of sensitization and irritation in an HRIPT at ten times the active clinical dose on more than 100 volunteers, supporting the “hypoallergenic” claim.
- Significant and progressive efficacy in a randomized, single‐blind, placebo‐controlled clinical trial on 60 volunteers: up to 24% reduction in overall skin pigmentation and 17% reduction in spot pigmentation at a concentration as low as 0.01%, without reaching an activity plateau at Day 84.

These results position Glucuronyl Glucosyloleanolate as a strong candidate to become a benchmark active ingredient in skin lightening and depigmentation. Its low effective concentration, patented status, dual mechanistic profile, and excellent safety record make it suitable for both cosmetic and medical applications. Future research will evaluate its anti‐aging and antioxidant properties in dedicated clinical studies to further expand and consolidate its field of application.

This work simultaneously validates the relevance of our proprietary AI‐assisted discovery platform and the effectiveness of the decision tree methodology, which is now positioned to identify new candidate ingredients addressing other market segments in search of more effective, safer, and scientifically grounded solutions.

## Author Contributions

Conceptualization: Y.Q.; Data curation: Y.Q.; Formal analysis: Y.Q.; Funding acquisition: Y.Q.; Investigation: Y.Q., M.G.; Methodology: Y.Q.; Project administration: Y.Q.; Resources: Y.Q.; Supervision: Y.Q., M.G.; Validation: Y.Q., M.G.; Visualization: Y.Q.; Writing – original draft preparation: Y.Q.; Writing – review and editing: Y.Q., M.G.

## Funding

This research was supported by the public service of Wallonia (“Service Public de Wallonie”, 8760).

## Ethics Statement

The authors confirm that the ethical policies of the journal, as noted on the journal's author guidelines page, have been adhered to. All procedures involving human participants were conducted in accordance with the Declaration of Helsinki (1964, as revised). The study protocol received approval from an independent ethics committee prior to initiation. The trial was conducted in compliance with Good Clinical Practice guidelines and applicable French regulatory requirements (Law No. 2004‐806, August 9, 2004). Written informed consent was obtained from all participants prior to any study‐related procedures. Participant anonymity was preserved throughout; all individuals were identified exclusively by assigned numerical codes in study documentation and data records.

## Conflicts of Interest

The authors declare no conflicts of interest.

## Supporting information


**Appendix S1:** supplementary information.

## Data Availability

All datasets generated and analyzed in the context of this study are available from the corresponding author upon reasonable request. Access to clinical trial data may be requested in writing from the corresponding author, subject to applicable data protection legislation.
