# Supplementary material for: Glucuronyl Glucosyloleanolate: A Safe and Potent AI‐Discovered Depigmenting Saponin
Source: J Cosmet Dermatol. 2026 Sep 23;25(10):e71210. doi: 10.1111/jocd.71210 (PMC13599461; doi:10.1111/jocd.71210)
Supplement: Supplementary file 1 [file JOCD-25-e71210-s001.docx]

***“*Glucuronyl Glucosyloleanolate: A Safe and Potent AI-Discovered Depigmenting Saponin”**

**Supplementary files**

**Patch test: Cutaneaous compatibility assessment of a cosmetic product under dermatological control. Patch test on 10 subjects during 48h**

| **Study aim** | To evaluate the cutaneous compatibility of a cosmetic product after single application under patc |
| --- | --- |
| **Experimental plan** | Monocentric - Simple blind / Shared panel |
| **Study center** | LABORATOIRE COSMEPAR 2 rue Alessandro Volta 44470 Carquefou, France |
| **Study dates** | From 11/19/2024 to 11/21/2024 |

Panel characteristics Healthy subjects were selected according to the inclusion and non-inclusion criteria

Volunteers are recruited without randomization.

Each volunteer included in the study completes a medical questionnaire and an informed consent form.

Study inclusion criteria:

- Healthy volunteer between the ages of 18 and 70;
- No history of intolerance or allergy to cosmetic products;
- Volunteer who has provided written, free, informed, and express consent;
- Volunteer who declares having health insurance;
- Volunteer who declares not currently participating in another clinical study that could interfere;
- Volunteer who certifies that the personal information provided to the investigator is accurate;
- Volunteer capable of following instructions and reliable in adhering to the protocol’s requirements (no language or intellectual barriers, etc.).

Non-inclusion criteria:

- Subject not responding to inclusion criteria;
- Subject being in exclusion period between 2 tests;
- Subject refusing to give his informed, written consent;
- Pregnant or nursing woman;
- Allergy or sensitivity to adhesive plaster and / or aluminium;
- Scars, active dermal lesions, tattoos, pigmentary marks, excessive pilosity on the study area;
- Hyper-irritable skin ;
- Skin pathologies ;
- Progressive or important diseases, or insulin-dependent diabetes;
- Topical or systemic treatments before and during the study: anti-inflammatory, anti-allergic, immune suppressors;
- Skin treatments;
- Excessive use: alcohol, tobacco, toxic substances

| **Subject** | Number | Women | Men | Ages | Skin type |
| --- | --- | --- | --- | --- | --- |
|  | 12 | 7 | 5 | between 20 and 70 years | Normal |

2. PRODUCT PRÉPARATION AND APPLICATION

Product preparation

Solid products: an enough quantity to fill the chamber is deposited.

Powder form products: an enough quantity of paraffin oil (according to the solubility) was added to obtain a texture adapted to the patch application and an enough quantity to fill the chamber is deposited.

Semi occlusive patch: quantity of product is placed on a Finn Chambers 8mm filter paper disc to be impregnated.

Product application

The methods of the application are gathered in the following table:

| **Application zone** | Back (scapular zone) |
| --- | --- |
| **Concentration** | Pure |
| **Patch type** | Semi occlusive patch (with filter paper) Finn Chambers on Scanpor (8 mm, 50 mm²) |
| **Patch duration** | 48 hours |
| **Application conditions** | Negative control: distilled water on filter paper- No washing and no product application took place on study area |

3. READING PHASE

The patches were removed 48 hours after the application. The study area was rinsed with distilled water and clinical readings were performed 30 minutes after the patch removal.

The subject was its own control. For each subject, the study was validated when the negative control (distilled water on filter paper or mineral oil on filter paper) caused no skin reaction.

Erythema and oedema were scored by the doctor or the trained technician according to a quotation from 0 to 3.

4. COMPATIBILITY ASSESSMENT

The subject irritation index (VII) was calculated for each subject by adding the scores obtained (erythema + œdema). The mean irritation index (MII) was calculated and corresponded to the mean of the subject’s irritation index (VII) reported to the total number of subjects who finished and/or validated the study.

The irritation indexes were determined according to the following formula:

| **Irritation index** | **Calculation** |
| --- | --- |
| VII | ∑ scores (erythema + œdema) |
| MII | ∑ VII / number of subjects |

The mean irritation index (MII) allows classifying the tested product according the following scale:

| **MII** | **Classification** |
| --- | --- |
| MII<0.25 | Non-irritant |
| 0.25 ≤ MII < 0.50 | Veryu slightly irritant |
| 0.50 ≤ MII  < 1 | Slightly irritant |
| 1 ≤  MII < 2 | Moderately irritant |
| MII  ≥ 2 | Irritant |

The table of readings performed 30 minutes after patch removal is presented in the appendix. The mean irritation index (MII) was calculated (0.0).

| **N°** | **Age** | **Sex** | **Phototype** | **Result 48h** | | | | |
| --- | --- | --- | --- | --- | --- | --- | --- | --- |
|  |  |  |  | **Blank** | | **GGO** | | **MII** |
|  |  |  |  | **Erythema** | **Oedema** | **Erythema** | **Oedema** |  |
| 1 | 65 | M | III | Neg | Neg | Neg | Neg | 0 |
| 2 | 67 | F | III | Neg | Neg | Neg | Neg | 0 |
| 3 | 46 | F | III | Neg | Neg | Neg | Neg | 0 |
| 4 | 68 | M | III | Neg | Neg | Neg | Neg | 0 |
| 5 | 51 | M | III | Neg | Neg | Neg | Neg | 0 |
| 6 | 53 | F | III | Neg | Neg | Neg | Neg | 0 |
| 7 | 50 | F | IV | Neg | Neg | Neg | Neg | 0 |
| 8 | 38 | M | III | Neg | Neg | Neg | Neg | 0 |
| 9 | 67 | F | III | Neg | Neg | Neg | Neg | 0 |
| 10 | 27 | F | IV | Neg | Neg | Neg | Neg | 0 |
| 11 | 29 | F | I | Neg | Neg | Neg | Neg | 0 |
| Conclusion: Mean Iriration index | | | | | | | | 0 |

**HRIPT:** conducted by Eurofins Evic Romania: ER 25/130-06 / 25-0648

This monocentric, single-blind Human Repeated Insult Patch Test with Challenge (HRIPT) was conducted at Eurofins Evic Romania (Bucharest) between October 13 and November 21, 2025, under coordination by Eurofins Evic France (Bordeaux). The study fulfills the requirements of the ANSM 'Test Clinique Final de Securite' framework for cosmetic ingredients and was approved by the Institutional Ethics Committee prior to initiation.

The primary objective was to confirm that repeated application of the investigational ingredient Glucuronyl Glucosyloleanolate (GGO) under maximized exposure conditions did not induce delayed contact sensitization in healthy adult subjects. A secondary objective was to characterize the general skin compatibility of the product throughout the study period.

The methodology is an adaptation of the Marzulli and Maibach HRIPT protocol for delayed contact hypersensitivity, as referenced in Contact Dermatitis (1976) and Regulatory Toxicology and Pharmacology (2008). The study design used each participant as their own control through paired application sites, with distilled water serving as a procedural negative control.

The study complied with the Declaration of Helsinki, ICH E6(R3) Good Clinical Practice guidelines (July 2025), EU Regulation 2014/536 on clinical trials, REGULATION (EU) 2016/679 on personal data protection, and the Colipa 1997 guidelines for human skin compatibility assessment.

The study synopsis is the following:

| **Study population** | Number of test subjects: 100 valid cases | |
| --- | --- | --- |
|  | Specific inclusion criteria: test subjects | |
|  |  | • aged from 18 to 70, |
|  |  | • female and/or male, |
|  |  | • with a phototype (Fitzpatrick): from II to IV, |
|  |  | • with all types of skin on body. |
|  | Specific non-inclusion criteria: test subjects | |
|  |  | • with personal history of adverse reaction to colophony, rubber, nickel, aluminum, patch materials, adhesive plaster, |
|  |  | • with family or personal history of atopy. |
| **Methodology** | Application of the investigational product, in healthy human subjects, by a technician, at the investigating center, to a skin site on the upper back, under maximizing conditions of exposure (under semi-occlusive patch) for a defined time.  Repeated applications 9 times to the same site (induction site) over a period of 3 consecutive weeks, period necessary to induce a possible allergy (induction period).  After a minimal 2-weeks rest period, with no product application, single application of the investigational product, under patch, to the induction site and to a virgin site and for a defined time, enabling to reveal a possible induced allergy (challenge).  Application in parallel of distilled water under the same type of patch (semiocclusive patch) and at the same defined times as the investigational product = control site.   Skin examination of the application site, before the 1st product application of the induction period and the application of the challenge phase and after each patch removal by the same investigator or technician, supervised by the investigator.   Reporting of the sensations of discomfort directly by the test subjects to the investigator or technician, during the study.  Assessment of the allergic potential – checking of the skin compatibility: | |
|  |  | • Accurate description of the skin reactions observed, |
|  |  | • Evaluation of the allergic reaction according to the ICDRG scale: ?+, (+), (++), (+++), |
|  |  | • Calculation of the percentage of reactive test subjects during the challenge phase and the induction period. |
| **Overall Conclusion** | Under the experimental conditions adopted:  - During the induction period, the repeated applications of the product Glucuronyl Glucosyloleanolate (diluted 0.1% with mineral oil), under semi-occlusive patch, on a panel of 102 test subjects with 100% all types of skin on body, induced no reaction of irritation.   - During the challenge phase, the single application of the investigational product, on a panel of 101 subjects, to the induction site and virgin site induced no allergic reaction.  Based on these results, the product has a very good skin compatibility and does not show a sensitizing effect. | |

**Investigational Product**

For the purposes of this study, the substance was dissolved at 0.1% (w/w) in mineral oil immediately prior to each application session and delivered using a calibrated single-use micropipette tip. This dilution was selected to achieve a maximizing exposure concentration appropriate for the HRIPT design, while remaining compatible with the patch vehicle. The 0.1% concentration in mineral oil also reflects realistic worst-case exposure in formulated cosmetic end products.

A semi-occlusive patch system was used: an absorbent Webril® support (400 mm² application area) held in position by a non-woven medical adhesive. Occlusion promotes percutaneous penetration of the test substance, thereby maximizing the probability of detecting any inherent sensitization potential. The control site received 160 µl of distilled water under an identical patch to account for any reactions attributable to the occlusion or patch materials rather than to the ingredient itself.

The product was stored at the investigating center under conditions defined by the sponsor, prior to any application. A retention sample was archived for at least 3 years post-study.

**Study population**

Test subjects were recruited from the standing volunteer panel maintained by Eurofins Evic Romania, which is continuously renewed and covers all social categories. Candidates underwent an initial medical examination and detailed medical/cosmetological questionnaire conducted by a general practitioner, then were further screened against the study-specific inclusion and exclusion criteria. All 108 included subjects were enrolled on the same day (D1).

A total of 119 subjects were screened; 108 met all criteria and were formally enrolled. An additional 19 subjects beyond the minimum required were enrolled at the outset to buffer against anticipated withdrawals. At study completion, 103 valid cases were available for irritation assessment and 101 for sensitization evaluation - both exceeding the sponsor-requested minimum of 100.

The table below summarizes the principal inclusion and non-inclusion criteria applied to this study. General non-inclusion criteria additionally excluded subjects who were legally detained, unable to provide emergency contact information, residing in residential care, or members of the investigating center staff.

| **Inclusion Criteria** | **Key Exclusion/Non-inclusion Criteria** |
| --- | --- |
| Age 18–70 years | Personal/family history of atopy |
| Male and/or female | History of adverse reaction to colophony, rubber, nickel, aluminium, patch materials |
| Fitzpatrick phototype II to IV | Documented contact allergy history |
| All skin types on body | Systemic retinoids within 6 months; antibiotics or antihistamines within 2 weeks |
| Healthy subject status confirmed by clinical exam | Vaccination within 2 weeks prior to study |
| Signed informed consent | Previous HRIPT within 3 months; pregnancy or breastfeeding; cancer treatment within 5 years |

The per-protocol population of 101 valid cases had a mean age of 56 years (range: 32–70). The panel was predominantly female (90%) and composed primarily of Fitzpatrick phototype III subjects (67%), consistent with typical Central/Eastern European cohorts. The phototype distribution and sex ratio were consistent across the included and valid case populations.

| **Parameter** | **Screened (N=119)** | **Included (N=108)** | **Valid cases (N=101)** |
| --- | --- | --- | --- |
| Age - mean (range) | - | 56 yrs (32–70) | 56 yrs (32–70) |
| Sex - Female / Male | - | 95 (88%) / 13 (12%) | 91 (90%) / 10 (10%) |
| Phototype I | - | 1 (1%) | 1 (1%) |
| Phototype II | - | 28 (26%) | 24 (24%) |
| Phototype III | - | 70 (65%) | 68 (67%) |
| Phototype IV | - | 9 (8%) | 8 (8%) |
| Withdrawals (personal reasons) | - | 7 | - |
| Protocol exclusions | - | 0 | - |
| Serious adverse events | - | 0 | - |

The HRIPT design comprises three sequential phases: an induction period (3 weeks), a rest period (minimum 2 weeks), and a challenge phase (1 week). The complete study ran from D1 (October 13, 2025) to D47 (November 21, 2025). The timeline is summarized in the following table:

| **Induction period: 3 consecutive weeks** | | | | | | | | | |  |
| --- | --- | --- | --- | --- | --- | --- | --- | --- | --- | --- |
| **Operations at the investigating center** | **Experimental times** | | | | | | | | |  |
|  | **D1** | **D3 - D5 - D8 -  D10 - D12 - D15 -  D17 - D19** | | | | | | **D22** | |  |
| Delivery of the informed consent form Signature of the informed consent form Checking of the inclusion and non-inclusion criteria | x |  | | | | | |  | |  |
| Clinical examination of the application sites and questioning of the test subject by the investigator or technician supervised by the investigator, before product application | n |  | | | | | |  | |  |
| Final inclusion | n |  | | | | | |  | |  |
| Application of the investigational product and  control under patch to the defined induction site | n | n | | | | | |  | |  |
| Removal of the patches at the investigating center |  | n | | | | | | n | |  |
| Clinical examination of the application sites and  questioning of the test subject by the investigator  or technician supervised by the investigator |  | n | | | | | | n | |  |
| Control of the observance |  |  | | | | | | n | |  |
| **Rest period: 2 consecutive weeks at least (4 weeks at the most)** | | | | No product application | | | | | | |
| **Challenge phase: 1 week** | | | | | | | | |  |  |
| **Operations at the investigating center** | | | **Experimental times** | | | | | |  |  |
|  |  |  | D43 | | D45 | D46 | D47 | |  |  |
| Clinical examination of the application sites and questioning of the test subject by the investigator or technician supervised by the investigator, before product application | | | x | |  |  |  | |  |  |
| Application of the investigational product and control under patch to the defined induction site and to a defined virgin site | | | x | |  |  |  | |  |  |
| Removal of the patches at the investigating center | | |  | | x |  |  | |  |  |
| Clinical examination of the application sites and questioning  of the test subject by the investigator or technician  supervised by the investigator | | |  | | x | x | x | |  |  |
| Control of the observance | | |  | |  | x | x | |  |  |
| *Investigator did not consider D39 necessary as a visit day | | | | | | | | |  |  |

**Induction Phase**

During the induction phase, the investigational product (0.1% Glucuronyl Glucosyloleanolate in mineral oil, 160 µl) and the control (160 µl distilled water) were applied simultaneously to two fixed, adjacent sites on the upper back, clearly demarcated on D1 by the study technician. Sites were selected to be free of scars, moles, freckles, and areas of friction with clothing.

Each product was pipetted using a single-use calibrated tip directly into the absorbent pad of the Webril patch, which was then applied and secured with the non-woven adhesive. Nine patch applications were planned across three consecutive weeks, following a Monday/Wednesday/Friday schedule:

- Patches applied on Mondays and Wednesdays were worn for 48 hours (±4 hours).
- Patches applied on Fridays were worn for 72 hours (±4 hours), covering the weekend.

Patches were removed at the investigating center. A skin examination was conducted by the same investigator or supervised technician within 15–30 minutes of each removal (or longer if post-adhesive redness persisted). Subjects were questioned at each visit regarding any sensations of discomfort felt while wearing patches at home. If strong discomfort occurred between visits, subjects were instructed to contact the center by phone; the patch could be removed early if warranted and a clinical examination would be scheduled.

The site location was precisely documented by the technician at the end of the induction phase to allow retrieval after the rest period.

**Rest Period**

Following D22 (the final induction patch removal), a rest period of a minimum of 2 weeks (maximum 4 weeks) elapsed with no product application. Subjects were instructed to report any skin reactions arising during this interval. This washout period is critical in the HRIPT design as it allows immunological sensitization, if initiated, to fully develop before re-exposure at challenge.

**Challenge Phase**

On D43 (the first day of the challenge phase), a pre-application clinical examination of both the former induction site and a newly designated naive (virgin) site was performed. The investigational product and control were then applied under patch to both sites simultaneously, using identical conditions to those used during induction. Patches were removed after 48 hours (±4 hours) at the investigating center on D45.

Post-removal skin examinations were conducted at D45 (15-30 min post-removal) and D47 (48h post-removal). The D46 visit was waived by the investigator as not required given the clinical picture. The two-site design (induction site and virgin site) is essential for interpretation: a reaction appearing exclusively at the induction site may indicate cumulative irritation rather than true sensitization, whereas identical or stronger reactions at the virgin site are indicative of an immune-mediated allergic response.

**Outcome Measurement and Scoring**

**Erythema Grading Scale**

All skin sites were assessed visually under standard daylight-equivalent illumination by the same investigator or supervised technician throughout the study. A 5-point erythema scale was applied:

- 0 - no visible erythema
- 1 - mild erythema (faint pink)
- 2 - moderate erythema (well-defined)
- 3 - severe erythema
- 4 - caustic effect (erosive and/or necrotic aspect)

If erythema scored 1 or above, the site was palpated to assess the presence of infiltration or oedema. Additional complementary reaction types were recorded using standardized codes (e.g., Oe = oedema, P = papules, V = vesicles, I/Pr = itching, D = desquamation, Dr = dryness, T = tape reaction).

**Sensitization Assessment - ICDRG Scale**

Allergic potential was assessed using the International Contact Dermatitis Research Group (ICDRG) grading scale, applied during the challenge phase. The scale is ordinal and qualitative, reflecting the clinical presentation of contact allergy:

| **Score** | **Definition** |
| --- | --- |
| IR | Irritation reaction |
| - | No allergic reaction |
| ?+ | Doubtful reaction (slight erythema only) |
| (+) | Weak positive reaction: slight erythema and infiltration with small papular elevations, no vesicle |
| (++) | Strong positive reaction: erythema, papules, vesicles, infiltration |
| (+++) | Extreme positive reaction: intense erythema, oedema, coalescent vesicles (bullae) |

Sensitization was defined as an ICDRG score of (+) or above at the challenge phase that was not observed or was less marked during induction - particularly when present at the virgin site. Erythema graded 2 or above during the challenge phase was considered a potential sensitization signal warranting re-challenge or complementary investigation.

A reaction was classified as irritant (rather than allergic) if it appeared predominantly at the induction site with higher intensity than at the virgin site and followed a pattern consistent with cumulative irritation.

**Compliance and Protocol Adherence Monitoring**

Subject compliance with study constraints was verified at the end of the induction period (D22) and at the end of the challenge phase (D47) via a standardized questionnaire. The investigator reviewed any deviations from protocol-mandated conditions and assessed their impact on result validity. Significant deviations - particularly those involving the use of interfering medications, sun exposure, or removal of patches before the required contact time - could result in exclusion from the valid case count.

**Protocol Deviations**

Three categories of minor protocol deviations were recorded. All were assessed by the principal investigator as having no impact on the validity of the study results, given the complete absence of any skin reaction throughout the study.

| **Deviation type** | **Description** | **Investigator assessment** |
| --- | --- | --- |
| Missing induction visit | 14 subjects completed 8 instead of 9 induction applications (1 missed visit each, for personal reasons) | Minor - no impact on results |
| Contact time deviation | 121 deviations (11.98% of total applications); range: –14h54min to +16h17min vs. target | Minor - no impact on results |
| Examination time deviation | 3 deviations (0.67% of total examinations); clinical exam at 11 min instead of 15 min post-removal | Minor - no impact, no reactions observed |

Fourteen subjects who missed one induction visit still received at least 8 patch applications, which is well within the accepted range to mount a sensitization response if the ingredient were capable of inducing one. Contact time deviations spanned a range of roughly -15h to +16h relative to targets; given the semi-occlusive patch design and the pharmacokinetics of percutaneous absorption, these variations are unlikely to materially alter the sensitization signal. Examination timing deviations of 4 minutes below the 15-minute minimum were similarly assessed as negligible.

**Results**

Induction Phase

During the induction phase, a total of 102 subjects received at least one post-application examination and were therefore evaluable for irritation potential. No erythema of any grade was recorded at any application timepoint (D3 through D22) for the investigational product site. No complementary skin reactions - including oedema, papules, vesicles, or itching - were recorded. The control site (distilled water) likewise showed no reactions, confirming the absence of any patch-material-related effect.

No application was discontinued due to irritation or sensitization at the induction site. No suspected allergic reactions were flagged during the induction phase, and no subject was excluded from the study on grounds of skin intolerance to the investigational product.

Challenge Phase

During the challenge phase, 101 subjects were evaluated. The investigational product was applied to both the previously treated induction site and a naive virgin site. No erythema, complementary skin reaction, or ICDRG-positive response was recorded at either site for any subject. No site reached an erythema score of 2 or above. The control site similarly showed no reactions.

No re-challenge (additional application) was deemed necessary. No confirmed allergic reaction was noted across the entire study population.

| **Phase** | **Population** | **Reaction type** | **Investigational product** | **Control (distilled water)** |
| --- | --- | --- | --- | --- |
| Induction | N = 102 | Erythema (E) | 0 / 0% | 0 / 0% |
| Induction | N = 102 | Complementary mention (M) | 0 / 0% | 0 / 0% |
| Challenge | N = 101 (induction + virgin site) | Erythema (E) | 0 / 0% | 0 / 0% |
| Challenge | N = 101 | Complementary mention (M) | 0 / 0% | 0 / 0% |
| Challenge | N = 101 | ICDRG scale (A) | 0 / 0% | 0 / 0% |

Adverse Events

One non-serious adverse event was recorded for subject ref. 08a (reported in Appendix 1), related to a missed D17 visit. No serious adverse events, no hospitalizations, no severe skin reactions, and no deaths occurred during the study. The study was not suspended at any point.

Overall Conclusion

Under the maximizing experimental conditions of this HRIPT, 0.1% Glucuronyl Glucosyloleanolate in mineral oil, applied under semi-occlusive patch, nine times over three weeks (induction), followed by a single challenge application to both the previously treated site and a naive virgin site; no irritation reaction was observed during induction and no allergic reaction was recorded during the challenge phase in a panel of 101-103 evaluable subjects.

The product Glucuronyl Glucosyloleanolate demonstrated very good skin compatibility and did not exhibit a sensitizing potential under these experimental conditions. These results are consistent with support for a 'hypoallergenic' marketing claim, subject to the standard qualification that the absence of sensitizing evidence in a controlled study panel does not categorically exclude rare and accidental sensitization events in very large consumer populations.

**Clinical trial**

The aim of this clinical study is to evaluate the depigmenting efficacy of the cosmetic active glucuronyl glucosyloleanolate versus placebo on a multi-ethnic panel (58 women, including 20 Caucasians, 18 Asians and 20 Africans) with pigmentation spots.

The product (active or placebo) is tested on 2 groups of volunteers for 3 months. The following analyses were performed on D0 (baseline), D21, D56 and D84:

- Acquisition of standardized photographs with VISIA CR5
- Chromametric measurement of 2 target hyperpigmented spots (Asian and Caucasian volunteers only) for depigmenting effect (ITA angle)
- Evaluation of skin lightening effect by chromametric analysis of the cheek (parameter L and ITA angle, Chromameter CR400, Konica Minolta)

The study was conducted under dermatological supervision, and volunteers completed a satisfaction questionnaire at the D84 visit.

Study start date (first visit D0): Monday November 4th 2024

Study end date (last visit D84): Friday February 14th 2025

PRODUCT UNDER STUDY: Active ingredient GGO 0.01% versus vehicle.

Instructions of use Apply the product (active or placebo) twice a day to the face for 3 months (84 days). Use of SPF50 sun cream in case of sun exposure. 1 bottle of 100 mL was given to each volunteer at the D0 visit. At the D56 visit, a second bottle will be given.

Inclusion Criteria

- GGO 0.01% group : 30 women
- 10 phototype IV (North African origin) or V (African origin)
- 10 subjects of Asian origin
- 10 subjects of Caucasian origin
- Vehicule : 30 women
- 10 phototype IV (North African origin) or V (African origin)
- 10 subjects Asian origin
- 10 subjects of Caucasian origin •
- Presence of hyperpigmented spots, lentigos or melasma (for Asian and Caucasian volunteers)
- Having received information on the study objectives and procedures, and being able to understand them,
- Have signed the consent form,
- Affiliated to a social security system.

Subject non-inclusion criteria

- Pregnant women, women wishing to become pregnant during the study or nursing mothers,
- Participants in any other clinical study evaluating cosmetics, drugs or medical devices, • Subjects with an autoimmune disease,
- Under concomitant treatment (or not discontinued for at least 3 months) with oral or injectable corticosteroids (inhaled corticosteroids are permitted, as well as topical corticosteroid therapy not involving the head or neck),
- Subjects with progressive cardiovascular, digestive, neurological, psychiatric, genital, urinary, hematological or endocrine disorders; in general, any serious and/or progressive systemic disease.

This study is carried out according to:

- good clinical practice (CPMP, July 1996)
- the law of December 20, 1988 (n ° 2004-806 of August 9, 2004)
- the declaration of Helsinki (1964) 5.2)
- Independence of the ethics committee.

Recruiting subjects: recruitment is carried out by the GREDECO laboratory on its own panel. The identity of each subject participating in the study remains confidential thanks to the use of an identification number.

All assessments were carried out on D0 (baseline), D21, D56 and D84.

Chromametric measurement of pigmentation in 2 target spots to assess the reduction in pigmentation of spots, the individual typology angle (ITA°) is measured to observe a modulation in pigmentation intensity. An increase in ITA° is observed if pigmentation intensity decreases (lightening of the analyzed area). At D0, the dermatologist selects 2 target spots which are photographed and their pigmentation is measured with the Chromameter at each visit.

Evaluation of skin lightening effect by chromametric analysis of the cheek (parameter L and ITA angle) Analysis of the L parameter and ITA angle enables us to observe any modulation of pigmentation intensity by variation in these parameters. An increase in the L* parameter and the ITA angle is observed in the event of a decrease in pigmentation intensity (lightening of the analyzed area).

2D VISIA photography 2D photographs are taken using VISIA to visualize the modulation of the parameters studied.

Dermatological tolerance: The tolerance of the gel will be assessed by the dermatologist. Throughout the study, the absence of adverse events is checked. In the event of an adverse event (pruritus, rash) reported by the volunteer, a clinical investigation is carried out by the dermatologist, and photographs may be taken. If these effects persist, the dermatologist remains in contact with the subject until the symptoms have completely subsided. All adverse events must be reported to the study sponsor within 48 hours and included in the study report. At each visit, a medical examination will verify the absence of adverse events.

Time-delayed series of measurements (D0 - baseline, D21, D56 and D84) are carried out by subjecting the same individuals to these different methods. The samples are therefore matched. The results are presented in tabular form (mean ± SD).

Intra-group statistical analysis (matched data) : The Shapiro Wilk normality test is performed on all the data distributions to determine whether a parametric or non-parametric test should be used. The ANOVA test with multiple comparisons will be used to compare different measurement times. For all statistical tests, an alpha risk of 5% is chosen (significance if p <0.05). All statistical tests are performed with GraphPad Prism 10.2.1 software.

A total of 58 volunteers were included in this study (2 out of study), with an average age for the GGO 0.01% treated group of 53.83 ± 9.19 (Min : 33 and Max : 70) and for the vehicle treated group of 53.14 ± 13.94 (Min : 25 and Max: 74).

No dermatological tolerance problems were observed. However, 2 subjects of Asian origin (one in each group), subject n°21 belonging to the GGO 0.01% treated group and subject n°40 belonging to the vehicle treated group, decided to stop the study after a few days (at D7) despite our recommendations, due to what they considered to be insufficient moisturizing of the face.

The clinical results are summarized in the two following tables for, the decrease of respectively, the overall skin tone (figure 2) and the pigmentation of the two targeted spots (table 12).

Figure 2: volunteer group II facial pigmentation evolution during clinical trial
